# Intergroup Cooperation in Shotgun Hunting Among BaYaka Foragers and Yambe Farmers from the Republic of the Congo

**DOI:** 10.1007/s12110-023-09448-0

**Published:** 2023-04-26

**Authors:** Vidrige H. Kandza, Haneul Jang, Francy Kiabiya Ntamboudila, Sheina Lew-Levy, Adam H. Boyette

**Affiliations:** 1 Department of Human Behavior, Ecology and Culture, Max Planck Institute for Evolutionary Anthropology, Deutscher Platz 6, 04103 Leipzig, Germany; 2 Faculté des Lettres, Arts, et Sciences Humaines, Marien Ngouabi University, Brazzaville, Republic of the Congo; 3 Department of Psychology, Durham University, Durham, UK

**Keywords:** Interethnic cooperation, Shotgun hunting, Social norms, Inequality, Foragers, Congo Basin

## Abstract

Whereas many evolutionary models emphasize within-group cooperation or between-group competition in explaining human large-scale cooperation, recent work highlights a critical role for intergroup cooperation in human adaptation. Here we investigate intergroup cooperation in the domain of shotgun hunting in northern Republic of the Congo. In the Congo Basin broadly, forest foragers maintain relationships with neighboring farmers based on systems of exchange regulated by norms and institutions such as fictive kinship. In this study, we examine how relationships between Yambe farmers and BaYaka foragers support stable intergroup cooperation in the domain of shotgun hunting. In the study village, shotgun hunting is based on a specialization-based exchange wherein Yambe farmers contribute shotguns and access to markets to buy cartridges and sell meat while BaYaka foragers contribute their specialized forest knowledge and skill. To understand how costs and benefits are distributed, we conducted structured interviews with 77 BaYaka hunters and 15 Yambe gun owners and accompanied hunters on nine hunting trips. We found that hunts are organized in a conventional manner within a fictive kinship structure, consistent with the presence of intercultural mechanisms to stabilize cooperation. However, because bushmeat demand is high, gun owners can gain significant cash profit, while compensating hunters only with cigarettes, alcohol, and a traditional hunter’s portion of meat. To level payoffs, hunters strategically hide kills or cartridges from gun owners to feed their own families. Our results illustrate how each group prioritizes different currencies (e.g., cash, meat, family, intergroup relations) and provide insights into how intergroup cooperation is stabilized in this setting. The example of this long-standing intergroup cooperative system is discussed in terms of its contemporary entwinement with logging, the bushmeat trade, and growing market intersection.

In the evolutionary social sciences, competition between groups has been proposed as key to the emergence of human cooperation (Bowles, 2009; Manson & Wrangham, 1991; Puurtinen & Mappes, 2009; Richerson et al., 2016). Theoretical and empirical work, for instance, suggests symbolic markers of group membership evolved to facilitate coordination within groups, and that such coordination would be selected for in contexts of conflict with other groups (Handley & Mathew, 2020; McElreath et al., 2003). Yet, markers of group membership, such as the often-arbitrary traits associated with ethnicity, can also facilitate intergroup cooperation if they are associated with specific roles or products, such as in contexts of trade (Lupo, 2006; Tucker et al., 2021). Moreover, most intergroup interactions, including those between self-identified ethnic groups, are peaceful and exhibit stable cooperation across political, economic, and social domains (Bunce & McElreath, 2018; Fearon & Laitin, 1996; Fry & Söderberg, 2014; Pisor & Surbeck, 2019; Rupp, 2011; Tucker et al., 2021).

According to Robinson and Barker (2017), intergroup cooperation is likely to be sustainable when groups exchange resources that each is more efficient at acquiring, leading to a division of labor. This is consistent with the idea that intergroup cooperation between human groups can effectively buffer against local resource shortfalls by spreading risk across the socio-ecological landscape (Cashdan, 1987, 2001a; Colson, 1979; Migliano et al., 2017; Wiessner, 1982). Along these lines, Pisor and Gurven (2016) found that, in lowland Bolivia, participants in an economic game were more likely to offer benefits to members of an out-group when they had a greater perceived need for nonlocal resources. Participants in this study also offered more to the out-group during the game if they had greater experience with out-group strangers (Pisor & Gurven, 2016). This latter finding is consistent with the general finding that repeated interaction, especially between specific known individuals from the out-group, increases the likelihood of intergroup cooperation by increasing confidence in generalized reciprocity (Robinson & Barker, 2017; Wiessner, 1982, 2002; Zori & Brandt, 2012).

When intergroup cooperation is based on exchange, the potential for exploitation can be high (Robinson & Barker, 2017), especially when groups vary in power (Cashdan, 2001b; Cochran & O’Connor, 2019; Ensminger & Knight, 1997). Cultural norms—or shared beliefs about appropriate behavior in specific contexts (Bunce & McElreath, 2017)—increase the stability of cooperation if they govern the terms of exchange (Fearon & Laitin, 1996; Robinson & Barker, 2017). However, theoretical models suggest that such norms need not dictate that groups receive equal benefits for the cooperative system to remain stable (Cochran & O’Connor, 2019). At the same time, another set of models and empirical tests suggests that intergroup cooperation can remain stable and profitable even for disempowered groups depending on their relative bargaining power (Bunce & McElreath, 2017, 2018), the strength of their commitment to their group identity, and their knowledge of how to navigate the other groups’ norms (Bunce, 2020, 2021). Altogether, these studies suggest that intergroup cooperation is a valuable means of human adaptation, but one with a different scale of coordination problems than those governing successful cooperation within groups (Pisor & Surbeck, 2019).

In this paper, we examine intergroup cooperation between a community of Ba-Yaka foragers and Yambe fisher-farmers in the northern Republic of the Congo. This case study represents one instance of a widespread pattern of intergroup relations between mobile forest specialists (referred to henceforth as “foragers”) and (mostly) Bantu-speaking horticultural peoples (referred to henceforth as “farmers”) in the Congo Basin (Boyette et al., 2022). Historically, Congo Basin foragers likely first encountered farmers when the latter migrated into the forest in multiple waves from around 2,500–5,000 years ago (Batini et al., 2011; Klieman, 2003). The Congo Basin tropical forest presents an array of ecological challenges, including high diversity and seasonality of edible plants and animals, foods low in micro-nutrients, and a high concentration of parasitic diseases (Bahuchet, 1988; Dormitzer et al., 1989; Guernier et al., 2004). As such, economic exchange based on resource specialization was most likely the driver of early cooperation as Bantu-speaking migrants adapted their savannah-based subsistence practices to the new forest ecology (see Boyette et al., 2022 for review). However, there was also profound intercultural exchange, as evidenced by the adoption of farmer languages by foragers (Bahuchet, 2012), and social and ritual dimensions commonly characterize Congo Basin forager-farmer relations today (Takeuchi, 2014). This broad pattern of interaction has likely remained stable for millennia, but historical and ethnographic research suggests highly dynamic relationships between specific villages, families, and individuals (Joiris, 2003; Takeuchi, 2014; Terashima, 1986). Using the framework of intergroup cooperation, in this paper we identify the specific social, cultural, and economic factors that support or undermine stable cooperation locally in order to better understand this dynamism at larger scales.

## Fictive Kinship Between Farmers and Foragers

One institution which patterns interethnic cooperation in the Congo Basin is that of fictive kinship between farmers and foragers. Fictive kinship organizes the exchange of forest- (e.g., honey, meat, medicine) and village-derived (e.g., manioc, iron) goods and connects individual foragers to individual farmers, and by extension links their families together (Joiris, 2003). Typically, the fictive familial relationship is inherited such that these linkages extend across generations. Different research traditions have emphasized different aspects of forager-farmer relations and the implications of conceiving of them as relations between kin. Economic or sociopolitical perspectives tend to portray foragers as dominated and denigrated by the farmers with whom they exchange because of the latter’s privileged position as intermediaries between the foragers and larger market systems (Bahuchet & Guillaume, 1982; Bailey et al., 1989; Grinker, 1994). Others have noted that foragers instead see themselves as “hunters” of farmer resources who resist coercion through mobility and an intimate knowledge of the forest (Lewis et al., 2005; Turnbull 1961). Indeed, forager individuals, families, and even whole communities have been known to break kin ties with farmer communities who treat them unfairly by moving into the forest for extended periods of time (Lewis, 2002). Elsewhere, foragers maintain ties despite alternative economic possibilities, emphasizing the multidimensionality of these intergroup relationships (Lupo, 2006). In this paper, we consider the importance of intergroup cooperation—specifically, in the context of shotgun hunting exchanges—as reflecting a resource diversification strategy which benefits both BaYaka foragers and Yambe farmers. Further, we examine how norms maintain interethnic cooperation in the face of threats to stability.

## Shotgun Hunting and Intergroup Cooperation

Hunting is a critical aspect of BaYaka subsistence and identity. Men regularly hunt, and their conversations are often dominated by hunting stories (Lewis, 2002). Provisioning one’s family—including through hunting—is an important responsibility for fathers (Boyette et al., 2020). Moreover, hunting is tied into a greater cosmology which connects hunting skill, reproduction, health, and social standing (Lewis, 2008). In the northern Republic of the Congo, men’s famous skill in hunting also means that game meat is one of many forest products BaYaka procure to exchange with farmers for cultigens and other non-forest resources.

In the context of this exchange, the use of specific hunting technologies has varied across space and time in this region, sometimes in response to market demands. Spear hunting is a historically stable means through which BaYaka hunt for direct consumption (Kitanishi, 1995; Lew-Levy et al., 2021, 2022). Net hunting was likely adopted from non-BaYaka for meat consumption and to produce duiker hides for markets in Europe (Bahuchet & Guillaume, 1982; Noss, 1997). Shotgun hunting began in 1899 when ivory traders provided guns to hunters to increase elephant hunting for the ivory trade (Bahuchet & Guillaume, 1982). As a highly efficient technology (Van Vliet & Nasi, 2008; Kümpel et al., 2009; Yasuoka, 2014; Duda et al., 2017), today shotgun hunting is central to provisioning bushmeat markets in logging towns (Barnes, 2002; Bowen-Jones et al., 2003; Duda et al., 2017; Fa et al., 2003; Lewis, 2002; Riddell, 2013; Wilkie & Carpenter, 1999; Yasuoka, 2014).

BaYaka’s limited participation in wage labor means that they cannot typically afford to own shotguns, nor can they regularly buy cartridges. In contrast, the farmers have deeper intersections into the market economy and skills in the management of money, leaving them in nearly exclusive control over this mode of production. Anecdotally, we have known multiple BaYaka men who have been gifted shotguns by outsiders such as researchers or filmmakers. In each case, these guns were taken by the men’s farmer fictive kin, who justified their actions with reference to debt (if they did so at all). Thus, it appears that farmers prefer to maintain control over shotguns, even though they could provide BaYaka an efficient, independent means of obtaining meat as well as cash from its sale. However, as our data will show, hunters are not powerless in their exchanges with farmers, and they assert control over how hunting returns are shared.

## Ethnographic Setting

Research for this study was conducted in a village along the upper Motaba River, which lies in the tropical forests of the District of Dongou in the Likouala Department of the Republic of the Congo. The primary ethnic groups living in the village are the Yambe and the BaYaka. According to Yambe elders, their ancestors migrated to Likouala where they met BaYaka in forest camps near the Motaba River. Thereafter, BaYaka hunters showed the Yambe one of the places richest in fauna at the edge of the Motaba River. Impressed, an elder Yambe decided to establish a new village there. Today, there are approximately 150 Yambe living in the village. The Yambe grow cassava, corn, plantains, and taro, among other smaller crops. They also fish using hooks, nets, and traps. Yambe women make palm oil and distill alcohol with maize and cassava. Many Yambe families have shotguns, and they typically recruit BaYaka hunters for hunting with shotguns. The interethnic cooperation in this labor exchange is the subject of this study.

The BaYaka population in the study village is estimated to be around 600 people. Their subsistence economy is typical of other BaYaka communities in the region. Men’s activities include hunting with spears, crossbows, traps, and snares, as well as collecting honey (Kitanishi, 1995). Women gather wild edible plants, including yams, nuts, seeds, leaves, and mushrooms. Men and women cooperate extensively in a variety of subsistence activities, such as clearing gardens, collecting liana fruits and caterpillars, and fishing (Jang & Boyette, 2021; Kitanishi, 1995). These gendered work activities are flexible; men sometimes gather and women can participate in hunting (Hewlett, 1991; Noss & Hewlett, 2001). The BaYaka interviewed for this study live in the village in December and January, and some return to the village for durations ranging from days to months throughout the year. From February to May, they primarily live in forest camps to fish, gather *Irvingia* seeds, and collect black pepper (*Piper nigrum*, or *ndongobela* in Yaka) for trade with Yambe.

As is typical of other such forager-farmer relations in the Congo Basin, the fictive kinship system in the village unites a BaYaka family with a Yambe family through a hereditary link. Intergroup cooperation ideally occurs only between individuals who are fictive kin. For the Yambe, these kin ties are inherited on either the maternal or paternal side. Fictive kin status can also be acquired through marriage among the Yambe, such that a woman has the right to employ the BaYaka family of her husband and vice versa. This system makes it possible for a Yambe to have a range of exchange partners among the BaYaka to call on for subsistence labor. As a consequence, an individual BaYaka can be claimed as kin by a large number of Yambe, but, in reality, this likely gives the BaYaka some ability to choose with whom they cooperate.

Many social and economic changes have occurred in the region during the past 20 years, driven by forestry and conservation groups. For instance, the village where this study was conducted is on the border between two forestry concessions assigned to different companies (Doremus, 2019). As a result, once only accessible by waterway (Kitanishi, 1995), the village is now directly connected to two logging towns by roads and thus accessible by car or motorbike. The road network has contributed to increases in population density through migration from workers of logging companies, conservation organizations, and bushmeat traders. The greater access to outside markets has led the Yambe to intensify several economic activities for which they traditionally rely on BaYaka labor, including cultivation of palm wine, production of *Irvingia excelsa* (or *payo*) cake, fishing, and the collection of palm nuts, palm oil, and black pepper. In addition, the Yambe have received cacao plants from the logging company to make cacao gardens. The cacao is then sold by Yambe for their own benefit. Critical to the current study, there has also been an increase in the recruitment of BaYaka to hunt using shotguns because of the growing demand for bushmeat and the increased access to local and regional markets (Kitanishi, 1995; Lewis, 2002; Yasuoka, 2006).

At the same time, conservation organizations have implemented strategies to protect endangered species through reduction of unsustainable hunting techniques and the creation of protected areas (Doremus, 2019; Riddell, 2013). There are now many legal restrictions around hunting, including limiting access to a large part of the forest, forbidding night hunting and the use of nylon or wire snares, and requiring hunting licenses (Ministere de l’Economie Forestiere, 2007). These restrictions, enforced by ranger patrols, deprive local populations of their traditional rights to the forest in the interest of wildlife conservation (Lewis, 2002; Riddell, 2013). Further, rangers reportedly tend to enforce BaYaka hunting while being complicit in shotgun hunts led by Bantu (Riddell, 2013), reinforcing the ethnic power structure established during the colonial period (Lewis, 2005). Moreover, logging companies can improve their image by becoming Forest Stewardship Council (FSC) certified through bolstering these conservation efforts (Doremus, 2019). Such certifications can negatively impact the health of families within their concession, likely because of the limits they place on subsistence hunting (Doremus, 2019). For instance, Doremus (2019) reports that, compared with those living outside FSC certified areas, BaYaka living within these areas reported eating less meat and cultivated starches in recent meals, were more likely to have experienced recent bouts of illness, and also showed greater income inequality between families. These observations are consistent with a prior survey finding that BaYaka living in areas within forestry or conservation zones complained about the reduction in meat in their diet due to restrictions and enforcement by ranger patrols (Riddell, 2013). The village where the current study was conducted is in an FSC zone. However, hunting is permitted within legally mandated areas reserved for local community use as long as restrictions against killing endangered species are respected (Ministère de l’Économie Forestière et de l’Environnement, 2010).

## Methods

Data for this study were collected from December 2019 to February 2020 in the study village, the nearby BaYaka neighborhoods, and in the surrounding forest during hunting trips. Permissions to conduct research in the Republic of the Congo were obtained from the Institut de Recherche en Sciences Exactes et Naturelles (IRSEN) and the Comité d’Éthique of the Insitut National de Recherche en Sciences Sociales et Humaines (INRSSH) in Brazzaville.

### Consent Procedures

Shotgun hunting is a very sensitive issue along the Motaba River in northern Congo because of proximity to Nouabale Ndoki National Park, and the increasing bushmeat and elephant poaching in the area. Neither ethnic group likes to discuss their hunting habits with firearms in fear of unjustified or excessive repercussions by law enforcement. Thus, obtaining the trust of the local community is essential to collecting information on shotgun hunting. Before starting data collection, a public meeting with the village community, including both BaYaka and Yambe, was organized. During this meeting, we explained in local languages (Yaka and Lingala) the aims of our research. We noted that we have seen more shotgun hunting in recent years and have heard many hunting stories in the area, and so we became interested in whether shotgun hunting has changed the way BaYaka people hunt and share game meat, and if their relationships with the Yambe farmers have also changed. We then explained that we wanted to speak with people privately about their experiences with shotgun hunting and that we would not share any private or sensitive information with anyone else in the community, or with the conservation organizations. Participants were informed they could withdraw from the study at any time. We made certain that people understood they could ask any questions about the research before deciding by consensus whether research could be conducted in the village and in the surrounding BaYaka neighborhoods and forest camps. Everyone agreed to be involved in the study. We then additionally obtained informed consent from each individual participant before conducting the interviews. At the end of the research, by way of compensation, gifts were given to all community members irrespective of whether they had participated in the study. While we describe some hunting activities that are technically forbidden in the region (i.e., night hunting with shotguns) for reasons of conservation, fauna management authorities disregard this practice in unprotected forest areas in recognition of local people’s right to hunt, fish, forage, and set up camp in these forests (for similar local interpretations of the law in Cameroon, see Ichikawa et al., 2017).

### Data Collection

Interviews were conducted using a semi-structured questionnaire designed to elicit BaYaka hunters’ and Yambe gun owners’ general experiences with arranging or participating in shotgun hunts, and about the relationship between the hunters or gun owners with whom they work. The different sets of questions asked of hunters and gun owners can be found in Table 1. For this part of the study, the first author and a local translator randomly approached BaYaka or Yambe men (e.g., in front of his house, on the way to the river, garden, or moving between houses) from 6:30 to 11:00 and from 13:00 to 16:00 each day during the study period. Once someone was encountered, he was asked if he remembered the study and the sensitive content, and if he wanted to answer our questions. Our final sample consisted of 77 hunters and 15 gun owners.

**Table 1:** Questions asked of BaYaka hunters and Yambe gun owners

BaYaka	Have you gone hunting with a shotgun for Yambe [farmer]?
	How many times? [not very often, often, very often]
	Who have you hunted for?
	Is he/she your fictive kin?
	How are you related?
	Did they propose the hunt to you?
	Why do you accept to hunt for them?
	Whose cartridges do you use?
	Who do you go with?
	What do you kill?
	What do you keep?
	Does the Yambe [farmer] know you keep meat?
	What do they give you for your work?
	How do Yambe [farmers] react after the hunt?
Yambe	Do you own a shotgun?
	How did you get a shotgun?
	How do you usually get cartridges?
	Which BaYaka have you asked to hunt for you?

In addition to the systematic interviews, we also draw on extensive participant observation in the village and in BaYaka camps. As part of this, the first author participated in nine shotgun hunting trips of different durations. During these trips, he documented the identity and role of participating BaYaka (e.g., hunter, hunter’s wife, hunter’s child), the number of cartridges used, the number and name of the animal species killed, the number and name of species eaten in the hunting camp, and the number of animals brought back to the village for the gun owner.

## Results

All hunters reported that it is always the Yambe gun owners who propose hunts. Typically, shotguns are owned by a Yambe male head of household. However, the household head’s wife or other family members can borrow the gun and organize a hunt, and he may rent the gun to other Yambe who do not own one but want to organize a hunt. Yambe try to time the organization of hunts so they can sell game to bushmeat traders who regularly come to the village, or at the local market that takes place every week for two days. The required shotgun cartridges are purchased from the market towns, typically during trips to sell meat. Although the cartridges can also be bought from traveling merchants who visit the village by outboard motor canoes twice a week, the price of those cartridges is twice as much as in town.

As we describe below, shotgun hunting exchange norms vary depending on how much game is sought by the gun owner. However, the general arrangement is that a Yambe community member proposes a hunt to one or more BaYaka hunters. When the gun is given to the hunters, they also customarily receive a small quantity of tobacco, which BaYaka claim gives them energy and aids their concentration in the hunt. Hunts are typically at night and a headlamp is used. As one of our interlocutors explained, “That is because in the night, the animals become confused when they see the light and they stand still, watching the headlamp light. Then, it is easiest for us to kill many animals.” Hunters are then entitled to the head, organs, and neck of each animal they kill—the “hunter’s portion.” The hunters are given little else for their work by the gun owner. In our sample, 85.7% of BaYaka hunters reported that they received tobacco or alcohol after the hunt, although 14.3% said they also received cassava tubers or flour.

This “hunter’s portion” comes from a norm followed in BaYaka society, typically for hunting by other means (e.g., with spears). However, in those traditional hunting contexts, the rest of the animal is shared within the BaYaka community. In the context of shotgun hunting, the person who provided the cartridges—typically the gun owner or his family—takes the rest of the animal. Thus, in shotgun hunting exchanges with Yambe, the gun owner can feed their family with the game or sell it for cash for other needs, but the hunter receives only the hunter’s portion, which is not enough to share. As we will see, because the hunter publicly receives this portion of meat plus tobacco and alcohol for their work but nothing more to share with their family, BaYaka take active strategies in private to improve their payoff from these exchanges.

### Shotgun Hunting in Village Social and Economic Life

The majority of BaYaka men interviewed (89.5%) confirmed that they hunted with shotguns for Yambe, although there was also diversity among these hunters in their experience. Most hunters (71.4%) claimed they went shotgun hunting for Yambe often (i.e., once a week), whereas 18.2% said they did not go often (i.e., just once or twice a month) and 10.3% said they went very often (i.e., twice or three times a week). Among these hunters, all considered the gun owners they worked for as fictive kin. Some of the hunters traced their inherited fictive kinship relationships specifically through their paternal (55.8%) or maternal (12.9%) family, and others through their in-laws (2.6%). However, others were more ambiguous and could not recall detailed information about their link to the gun owner (28.6%).

Most Yambe adults participate in shotgun hunting exchanges. The 77 hunters we interviewed named 32 Yambe gun owners for whom they worked. The number of times individual gun owners were named ranged from only once to as many as 15 times. Conversely, gun owners reported having worked with between one and nine BaYaka hunters (mean = 5.43, SD = 2.0), suggesting some specialization among the Yambe in shotgun hunting exchange. Among the 15 adult Yambe men we spoke with, four reported that they did not own a gun but still organized hunts. Only one farmer reported that he never proposed shotgun hunting to the BaYaka hunters because he focused on fishing.

During the study period, we observed that hunting arrangements were a regular subject of discussion. BaYaka would talk about when and how the gun and cartridges will move from the hands of the gun owner to the hands of the hunter, and whether the hunter must retrieve it or if the Yambe will bring it to their camp—typical of larger hunts in which the gun owner has invested relatively more resources (see below). All Yambe and BaYaka with whom we spoke conceived of the relationship as a division of labor, reporting that both groups worked together in shotgun hunting. From the Yambe perspective, they depend on the BaYaka’s expert knowledge of the forest. For example, one prominent Yambe community member explained:

The BaYaka are the masters of the forest. They possess forest knowledge better than us Yambe. For them, the forest is like their house, they know every area where they can find animals at any time. This is why we give them guns every time we have cartridges, because we cannot walk in the forest very well like them.

The Yambe view their role in the relationship as providing access to the guns and to markets to buy cartridges and forms of non-cash compensation. On one hand, BaYaka typically lack numeracy and thus are vulnerable to exploitation should they attempt to sell meat themselves for cash. On the other hand, because of greater Yambe integration into markets and their greater need for cash to pay for such things as school fees, it benefits the Yambe if BaYaka do not acquire market competencies. As evidence of Yambe resistance to BaYaka earning cash, when hunters have attempted to sell meat in the village that they caught by other means (e.g., traps or spears), Yambe attempt to barter for the meat with other goods, such as agricultural produce and tobacco. Similarly, if a BaYaka were to try to sell game killed with a shotgun in the village, they would be asked from whom they got the gun and the cartridge because normally it is only through a Yambe that they would have received the gun, and it is therefore the gun owner who would have the rights to sell the meat. One gun owner described the relationship as such:

Shotgun hunting is the way that we are helping each other, because I will not eat this meat by myself—the BaYaka hunter will also eat the same meat. For example, say I just came back from the river to collect palm wine but I have nothing to eat with my family. If I had one or two cartridges in my house, I should automatically ask a BaYaka to go shotgun hunting this evening with a headlamp. Then, we all will expect to eat meat tonight or tomorrow. Another thing is that when the hunter brings a large quantity of meat, at this time we will take a quantity to eat, and I will sell the remaining quantity of meat because I must send money to my sons who are studying in the town.

Despite this view that they are “helping each other,” there is tension around Yambe efforts to keep control over money and market access. For instance, an elder BaYaka hunter recounted the following attempt to bypass the Yambe in shotgun hunting:

One day I found two big elephants’ tusks during shotgun hunting. I brought these elephants tusks to the gun owner. I told him to sell these elephants’ tusks for me and then buy a gun. Until now, I never received any benefit from the gun owner for my elephants’ tusks. At that time, I gave all the animals I had killed to the gun owner and my elephants’ tusks because I was expecting to get a gun.

Nevertheless, most BaYaka hunters (97.4%) reported that they accepted proposals to hunt for the Yambe because it was a way to get food for their families. Specifically, hunters keep a proportion of their kills without informing the gun owners so that they have something to share with their community. For instance, one hunter explained:

One day the chief provided me one cartridge and the gun. I went hunting at night and I killed a *mosome* (Peter’s duiker). I brought the mosome to my house and we ate the entire animal with my family and in-laws. Then, in the morning I went to the gun owner and said that I shot one mosome but I did not succeed to find the animal; I have to go during the day to check very well in the area where I shot. In the evening, I told the gun owner that I saw the blood but the animal had escaped.

Such maneuvers are common. Across the nine hunting trips joined by the first author, there were no missed shots, but as can be seen in Table 2, an average of 72% of kills were delivered to the gun owner. Moreover, on these trips, the BaYaka hunters and their families tended to eat the larger animal species more than the medium-sized species. Table 3 presents the relative sizes and weight range of each species. Converted to kilograms of uncooked meat, the total average weight of game given to the gun owner represented an estimated 312.5 kg, consisting of medium, large, and a few very large species. In comparison, even though they kept only an average of 38% of animals killed, hunters and their families tended to keep the larger species—in this case, an estimated average of 773.5 kg of uncooked meat across the nine hunts.

**Table 2:** Summary of nine observed hunting trips

Hunt No.	Type	Days	Hunters	*N* of BaYaka in hunting party	Animals killed	% of animals given to gun owner	Estimated average weight (kg) of meat eaten by hunters	Estimated average weight (kg) of meat given to gun owners
1	Short, multi-night	5	3	12	5	60	197	37
2	Short, multi-night	4	3	10	5	60	165	38.5
3	Short, multi-night	4	4	12	7	57	282	91
4	Short, multi-night	4	3	9	6	67	101.5	119.5
5	Short	1	1	1	1	100	0	4.5
6	Short	1	1	1	2	50	19.5	4.5
7	Short	1	1	1	1	100	0	4.5
8	Short	1	1	1	2	50	8.5	4.5
9	Short	1	1	1	2	100	0	8.5
Total		22	18	48	31		773.5	312.5
Mean, SD		2 ± 1	2 ± 1	5 ± 4	3 ± 2		86 ± 99	35 ± 40

**Table 3:** The animal species killed during the nine observed hunting trips. Relative sizes are based on Duda et al. (2017) and weight ranges on Stuart and Stuart (2006)

Vernacular names	English names	Scientific names	Size	Weight range (kg)
*Bea*	Giant forest hog	*Hylochoerus meinertzageni*	Very large	130–235
*Djombi*	Black-fronted duiker	*Cephalophus nigrifrons*	Large	13–16
*Mosome*	Peter’s duiker	*Cephalophus callipygus*	Large	15–24
*Ngouomu*	Bay duiker	*Cephalophus dorsalis*	Large	19–24
*Mbuli*	Sitatunga	*Tragelaphus spekii*	Very large	55–115
*Senge*	White-bellied duiker	*Cephalophus leucogaster*	Large	12–15
*Bemba*	Yellow-backed duiker	*Cephalophus sylvicultor*	Very large	45–80
*Ndjoua*	Red river hog	*Potamochoerus porcus*	Very large	45–115
*Tamba*	Agile mangabey	*Cercopithecus agilis*	Medium	4–13
*Gweti*	Mustached guenon	*Cercopithecus cephus*	Medium	3–5
*Mboloko*	Blue duiker	*Cephalophus monticola*	Medium	3–6

Although the BaYaka also hunt using methods that do not require working with the Yambe, many hunters during the interviews said that in the present day, they eat mostly animals killed with guns (*Kenekene wa mo woupa bo ba dzia niama mondoki*). This is because hunting with guns is easier and faster than spears or traps. Thus, while Yambe attempt to maintain control over the distribution of cash and market-derived products, the BaYaka attempt to maintain control over the distribution of game from the forest—each group applying their own norms of fairness to the exchange.

The Yambe are not ignorant of the possibility that hunters deceive them since hunters are not uniformly careful. For instance, occasionally conflicts lead to gun owners no longer working with a hunter in shotgun exchanges. This cessation of cooperation occurs when the gun owner finds irrefutable evidence that the hunter had killed an animal without giving the gun owner anything. For example, a gun owner explained to the first author that he had caught his BaYaka kin in a lie after returning from the hunt. The gun owner had given the hunter four cartridges, and after spending three nights in the forest, the hunter came back, reporting that he had missed every shot. While the hunter was talking, the gun owner noticed the blood stains on the hunter’s shirt. The gun owner then asked the hunter why he had blood on his shirt, if he had not killed any animals. The hunter stayed quiet, unable to justify the animal blood on his shirt. In response, the gun owner refused to recruit him to hunt in the future. However, the gun owner did not sever his relationship with the hunter entirely; he continued to seek his cooperation in other work (e.g., procurement of palm wine, palm oil, black pepper; garden clearing).

### Variation in Hunts and Their Norms

According to our interlocutors’ reports and our own observations, there are conventional stages to organizing hunts that vary according to the type of hunt arranged, from single night hunts to weeks-long trips (Table 4). How BaYaka overtly and covertly benefit from shotgun hunting depends on the arrangement. Specifically, the way of asking a hunter and handing over the gun is more elaborate when the gun owner wants to arrange a longer trip, reflecting the greater investment from both parties.

**Table 4:** Summary of key features of types of hunts

Type of hunt	No. of cartridges	No. of guns	Hunting party	Given to hunter according to norm	Meeting type	Maximum timespan	Benefit to gun owner	Benefit to hunter
Short hunt	≤ 3	1	Lone hunter	Cigarettes (sometimes)	Informal	Overnight or morning	Fresh meat for immediate consumption	Fresh meat for immediate consumption; meat to share
Short, multi-night trip	≤ 9	1	2–3 families	Cigarettes, alcohol	Formal	Three to five nights	Fresh meat for immediate consumption; meat to sell	Fresh meat for immediate consumption; meat to share
Long, multi-night trip	≤ 75	2–3	3–4 families	Cigarettes, alcohol, cassava flour, batteries, market goods (e.g., clothes, shoes, radios)	Formal	One to three weeks; gun owner accompanies	Fresh meat for immediate consumption; meat to sell	Fresh meat for immediate consumption; cartridges; market goods (promised but not always received)

*Short hunts*. Short hunts are typically intended to procure meat for immediate consumption within the gun owner’s household. In such cases, the hunter will go out, typically at night, and return that night or the following morning. These arrangements are fairly casual. For instance, if a BaYaka hunter visits the gun owner’s area in the village during the afternoon for other reasons, such as to see his wife, who is working for his or another household, the gun owner might use this opportunity to call the man to his house and propose a shotgun hunt. Other times, the gun owner may ask someone such as his son, daughter, or another BaYaka to call on the hunter before nightfall to come to his house to ask him to go hunting. In such cases, the gun owner will give the hunter a gun and one or two shotgun cartridges immediately, sometimes also with a cigarette.

At this moment, the BaYaka hunter must decide whether to go shotgun hunting with a headlamp if he has batteries or to go to sleep and then wake up before sunrise to hunt. If he shoots animals during the night, he will typically return home to sleep and bring the game to the gun owner in the morning. If the hunter leaves early the next morning instead, he’ll typically return late in the afternoon. Before bringing the fresh game to the gun owner, the hunter will take the hunter’s portion. As can be seen in Table 2, however, in two out of five short hunts observed during the study, the hunter kept one of the two animals killed. There is no normative gift or meeting after these hunts. Nevertheless, sometimes hunters may ask the gun owner for a cigarette, which may or may not be given.

Hunt 9 in Table 2 is an example of a short hunt. The first author and his translator accompanied one hunter on a trip to hunt blue duiker (*A me dua bokengoli ba mboloko*). Together, we left the village around 07:00, walking on the trail without talking. The hunter stayed alert, at one point identifying two species of monkey in the canopy by their voice (*Cercopithecus nictitins* or *koyii* and *Cercopithecus cephus* or *gweti*), but he was not interested in monkeys at this point. While we kept walking deeper into the forest, the hunter saw tracks of a blue duiker (Table 3). He asked us to stand and wait for him; then he moved about 50 m away from us and started to call animals by imitating a leopard (*ndzango wa ebongo*). A few minutes later, he shot an adult male blue duiker. Because we were far from the village, the hunter said: “We must return, and I will shoot a monkey on the way back.” In the village, the hunter gave both the duiker and the monkey (*gweti* or moustached guenon, Table 3) to the gun owner, keeping only the hunter’s portion.

*Short, multi-night trips*. In the case of trips for which hunters stay in the forest for multiple nights, typically less than a week, the gun owner calls the BaYaka hunter or hunters with whom he has a fictive kinship link to his house. Serious and respectful, the gun owner sits with the hunters, shares a cup of distilled alcohol or palm wine, and proposes a trip. At this time, the gun owner will explain that he wants a hunt of a certain number of days so the hunting party will return with the expected bushmeat in time for the gun owner to take it to the market. The hunter will then be given a gun and usually three to five cartridges, though the hunter may receive up to nine cartridges.

During short trips, the hunter will stay three or four nights in the forest before returning to the village with smoked meat. For these trips, hunters are usually accompanied by other BaYaka, with a group often composed of two to four men, their wives, and children. The hunters we interviewed told us that all BaYaka men who are involved in shotgun hunting are skilled hunters. Thus, if one of them is offered the opportunity for a multi-night trip by a gun owner, the lead hunter calls his closest friends and brothers-in-law accompany him. Then, each time a BaYaka hunter receives a gun there is an opportunity for sharing and assurance they will eat meat.

According to BaYaka, the most commonly targeted animals on these hunts are antelope and forest pig, in contrast to single night hunts when smaller game are targeted. Their explanation for these target priorities is that the use of cartridges against small animals such as blue duikers and monkeys is wasteful on these trips when they typically travel farther from the village. Blue duikers and monkeys are easily shot near the village.

During these hunts, fresh meat killed by the hunters is cooked and shared among the BaYaka in the forest. The meat that will be brought back to the gun owner is smoked. Some of the smoked meat may be consumed by the gun owner’s household, but most is sold to bushmeat traders who visit the village by motorbike, boat, or car. Alternatively, the gun owner himself will travel to other villages to sell the meat.

As an illustration of these short, multi-night hunts, the first author and his translator accompanied three hunters (two adults and one youth), their wives, and three children (Hunt 4, Table 2). We stayed in the hunting forest camp for three nights. During the first day in the forest camp, we left at 16:00 with four cartridges and two headlamps. The targeted animals were bay duiker, Peter’s duiker, yellow-back duiker, and red river hog. The hunters saw tracks of a group of red river hogs and yellow-back duiker, and they told me that the group of red river hogs was moving to the swampy area in the same direction we were walking. While the sun set and the forest became dark, we stood, and the hunters smoked cigarettes.

When they finished smoking, the lead hunter (the one recruited by the gun owner) asked us to stay quiet while he turned on the headlamp and put the cartridge in the gun. Then, alone, he walked for almost twelve minutes with the headlamp before we heard the first shot. We heard the second and third shots thirty and sixty minutes later. He then came back to us carrying one red river hog and he asked the other hunters to go collect the other two animals (a second red river hog and one *ngouomu*, bay duiker). That night when we returned to the camp carrying three animals, there was a big party because everyone knew that they would cook and eat the red river hog. The lead hunter gave the women half of the red river hog to cook. The day after, the hunters stayed in camp all day while their wives smoked the remaining meat. That day, the hunter left the camp at 18:00 with a headlamp and three cartridges. This second night, the hunters shot two *bemba* (yellow-back duiker) and one *mboloko* (blue duiker). They shared an entire yellow-back duiker among themselves. During the third night, the hunters and their wives smoked the rest of the meat to take back to the village.

After returning from a multi-night hunting trip, the hunter goes to the gun owner’s house, puts the meat in the kitchen, and waits in the yard with the men who accompanied him on the hunt. Then, the gun owner asks his wife or child to bring a container of 5 or 10 L of palm wine to give to the hunter, often with tobacco. Consistent with BaYaka sharing norms, although only one hunter was recruited for the work, his companions will share the palm wine and cigarettes that he received as a gift for the work.

*Long, multi-night trips*. Yambe organize shotgun hunting trips between one and three weeks long for which they recruit multiple hunters. We were not able to observe any of these longer hunts first-hand during the study period. For these, the gun owner invests significantly more than they do in shorter hunts, and usually one gun owner accompanies the hunters to control the number of cartridges used and to ensure that the meat is well smoked. Gun owners told us that for these trips, two or three guns are necessary, and they use between 25 and 75 cartridges. The gun owner also provides cigarettes for the hunters, cassava flour for the group to eat with game caught during the trip, and batteries for headlamps. Moreover, additional compensation is offered contingent on successful sale of the meat.

Alongside the greater economic investment, there is also an elaboration of the arrangement process. The gun owner first calls the hunters from among his BaYaka fictive kin for a meeting around a cup of palm wine or maize liquor. During this meeting, the gun owner seeks confirmation from each hunter that they will not be working for another Yambe or otherwise occupied. The gun owner will also inform the hunters of the details of the hunt, such as the amount of tobacco he’ll bring, the quantity of cassava flour, the total number of cartridges, when he’d like to go, and where the hunt will take place (the Yambe and BaYaka have familial hunting rights to specific parts of the forest). The gun owner also offers to buy clothes, shoes, radios, or other market-sourced goods as compensation for hunters. The offer must be reasonable because the gun owner requires each of the men to ensure his return on investment. For example, on one occasion a gun owner called three BaYaka to his house, and told them, “The day after tomorrow we will go hunting. You must let me know if you will come because I do not want to postpone my schedule or go to your house to recall you. I already have cassava flour, cigarettes, and batteries.” The BaYaka response is usually “No problem, we agree” (*Mondo te, bouse mo ndinga*). During these hunts, the gun owner gives each hunter two to four cartridges each day and he or she stays in the forest camp to smoke meat while the hunters pursue game.

At the end of the hunting trip, all smoked meat is brought to the gun owner’s house. The gun owner’s wife or child brings 20 or 25 L of palm wine, and sometimes 1 L of corn alcohol, and cigarettes to give to the hunters. The gun owner will remind the hunters that he will bring the things that he promised as payment after selling the bushmeat at the market. In this situation, BaYaka hunters eat the hunter’s portion of the fresh game every day in the forest, as well as portions of the smoked meat with authorization from the gun owner, usually in the morning. However, they are not able to easily hide game for themselves because of the presence of the gun owner. Nevertheless, BaYaka hunters report that they always keep two or three cartridges during these trips to use at a later hunt. They hide these cartridges in the forest to be retrieved later, telling the gun owner that these were missed shots. Thus, going on longer hunts, with more cartridges being used, offers the BaYaka a chance to stockpile cartridges for later use.

## Discussion

In this study, we have described how interethnic shotgun hunting exchanges occur in one Congo Basin village. These exchanges, for the moment, are a central feature of the economies of both BaYaka and Yambe communities and represent an example of stable intergroup cooperation. However, this case study also reflects the reality of cooperation across cultures: what looks like stability at one level is a dynamic series of negotiations between individuals who may not share the same conception of what cooperation should look like. The features of this system support theoretical models of intergroup cooperation.

Prior research indicates that exchange based on resource specialization stabilizes cooperation because it can help buffer risks of resource shortfall (Cashdan, 1987; Robinson & Barker, 2017), something especially important in complex ecologies such as the Congo Basin (Boyette et al., 2022). Our informants were clear that they exchanged based on specialized access to different resources, with the BaYaka regarded as forest specialists and the Yambe holding control over access to shotguns and cartridges procured from markets. These partnerships were multidimensional since Yambe and BaYaka cooperate in the procurement of several different local resources and also share some ritual and social obligations. As such, even if one side of the partnership perceives defection from the other—as we saw in the anecdote above describing a hunter being caught with blood on his shirt—the possibility of cooperation in other domains remains.

Cooperation in this system was further organized around a set of norms, consistent with research on the stabilization of intergroup cooperation (Bunce & McElreath, 2018; Fearon & Laitin, 1996; Robinson & Barker, 2017). As we described, two norms seem to stabilize intergroup cooperation between BaYaka and Yambe: norms which regulate relationships, and norms which regulate activities. First, exchanges were organized by a fictive kinship system, with all BaYaka hunters reporting that they hunted for their Yambe kin. This fictive kinship system establishes rights and responsibilities between partners, including an expectation that intergroup cooperation is exclusively through these fictive family ties, and is maintained by generalized reciprocity across repeated interactions—a mechanism that helps stabilize cooperation generally (Pisor & Gurven, 2016; Robinson & Barker, 2017). Second, all shotgun hunts were organized according to specific norms of interaction and exchange. As we’ve shown, gun owners always approach BaYaka, and BaYaka can consistently claim tobacco before the hunt and the hunter’s portion of the meat after the hunt. The receipt of alcohol and market goods is also common, though dependent on the length and intensity of the hunt. Altogether, these norms structure coordination in ways that allow gun owners and hunters to gain benefits from collective action (Ensminger & Knight, 1997).

Models have also shown that intergroup cooperation can stabilize at an equilibrium that benefits one group over the other (Cochran & O’Connor, 2019). There is no doubt that the system studied here appears, from a market perspective, unfair to BaYaka. Farmers may earn from 4€ for a blue duiker to 55€ for a red river hog, whereas one shotgun cartridge costs between 0.76€ and 1.06€. Tobacco costs much less. Thus, Yambe are making roughly as much as a 550% profit on the exchange, with little if any cash proceeds returning to BaYaka. Importantly, however, meat is a highly desired food that is widely shared among Congo Basin foragers and has important nutritional value (Bahuchet, 1990; Dounias & Ichikawa, 2017; Kitanishi, 1998). Hunters’ wives and families complain if men bring meat into camp that will not be shared but given to the gun owner (Lewis, 2005; Riddell, 2013). In our results, nearly all BaYaka hunters reported that they hunt to provision their families. We also found that most BaYaka hunters hide the majority of meat by weight for immediate consumption, or they hide cartridges for future hunting. Based on weight ranges reported in Table 3, each cartridge could be worth a 235 kg wild hog. In other words, BaYaka strategically and actively level what they perceive as payoff inequality. Thus, these findings suggest that relatively covert mechanisms for equalizing benefits may help stabilize intergroup cooperation in contexts of resource exchange. In this case, one group provides the means of production to the other but cannot easily police its use, even when they know it is used in ways outside of the agreed-upon exchange (i.e., gun owners cannot typically prove a shot was *not* missed).

By taking advantage of the better conditions created by logging companies, such as roads, increasing population density, and the market, our findings show that gun owners often initiate shotgun hunting because of the demand for bushmeat. This intensification has led to more—rather than less—intergroup cooperation. As we’ve shown, BaYaka hunters’ expert knowledge of the forest gives them significant bargaining power in the relationship (Bunce & McElreath, 2017). However, over time, these dynamics may shift. Wilkie and colleagues (2000) showed that in the absence of alternative sources of income, households in the forest significantly increase hunting for the market if they have access to roads and transportation. Construction of forest roads has been shown to lead to increased bushmeat hunting and steep declines in wildlife populations (Kleinschroth et al., 2019; Nasi et al., 2011; Riddell, 2013; Wilkie et al., 2000). Moreover, households that are not employed by the logging companies but that have access to the market (such as the Yambe) eat no more bushmeat, but they hunt more intensively and sell a higher proportion of game meat on the market (Wilkie et al., 2000). In sum, the bushmeat trade feeds the logging industry, which is depleting the forest with which BaYaka maintain their autonomy, livelihood, identity, and in the case of interethnic cooperation, their bargaining power (Lewis, 2005).

In sum, this case study suggests that models highlighting the impact of power imbalance between two groups on the relative benefits of stable cooperative interactions are likely applicable to forager-farmer interactions in the Congo Basin. However, this study also highlights the challenges in discerning the relative benefits across different currencies and in a shifting economic context. For the time being, BaYaka hunters are managing to redistribute benefits in ways that may be more acceptable to their families and to communities with whom they share. However, as game populations and habitats diminish and needs for money for subsistence increase, the stability of the current cooperative system will be challenged.

## Conclusion

Our findings show that cultural institutions and compatible goals support productive interethnic cooperation in the domain of shotgun hunting. We also found that while the benefits of shotgun hunting appear unequal at first glance, the covert actions of BaYaka hunters bring the payoff structure of shotgun hunting in line with BaYaka sharing norms. Together, these findings highlight one way in which communities maintain long-term intergroup cooperation through norms and trade specialization in risky ecologies (Pisor & Gurven, 2016; Robinson & Barker, 2017).

In our future work, we hope to track a range of dynamic variables which may further help or hinder intergroup cooperation. First, we need to better understand how the cultural institutions that regulate intergroup cooperation are transmitted from one generation to the next (Bombjaková, 2018; Bunce & McElreath, 2017; Pope-Caldwell, Lew-Levy, et al., 2022). Specifically, it would be interesting to investigate whether success biases play a role in the spread of hiding kills or cartridges and other behaviors which lead to higher payoffs for BaYaka (McElreath et al., 2003). Second, as we’ve outlined, the increase of shotgun hunting is the consequence of social and economic changes related to logging and conservation. Prohibitions around night hunting and trapping have indirectly reinforced shotgun hunting cooperation between Yambe and BaYaka because BaYaka have limited alternative means to hunt for themselves. In the absence of alternative income, shotgun hunting can only increase, leading to steep declines in wildlife populations (Kleinschroth et al., 2019; Nasi et al., 2011; Riddell, 2013; Wilkie et al., 2000). Understanding how market integration, increasing demands for bushmeat, and declining wildlife affect forager-farmer relations is, thus, an important area for enriching our understanding of intergroup cooperation in times of shifting social and ecological landscapes.

## References

[CR1] Bahuchet, S. (1988). Food supply uncertainty among the Aka pygmies (Lobaye, Central African Republic). In I. de Garine & G. A. Harrison (Eds.), *Coping with uncertainty in food supply* (pp. 118–149). Oxford University Press.

[CR2] Bahuchet S (1990). Food sharing among the pygmies of Central Africa. African Study Monographs.

[CR3] Bahuchet S (2012). Changing language, remaining pygmy. Human Biology.

[CR4] Bahuchet, S., & Guillaume, H. (1982). Aka-Farmer relations in the northwest Congo Basin. In R. B. Lee & E. Leacock (Eds.), *Politics and history in band societies* (pp. 189–210). Cambridge University Press.

[CR5] Bailey RC, Head G, Jenike M, Owen B, Rechtman R, Zechenter E (1989). Hunting and gathering in tropical rain forest: Is it possible?. American Anthropologist.

[CR6] Barnes RF (2002). The bushmeat boom and bust in West and Central Africa. Oryx.

[CR7] Batini C, Lopes J, Behar DM, Calafell F, Jorde LB, Van der Veen L, Comas D (2011). Insights into the demographic history of african pygmies from complete mitochondrial genomes. Molecular Biology and Evolution.

[CR8] Bombjaková, D. (2018). *The role of public speaking, ridicule, and play in cultural transmission among Mbendjele BaYaka forest hunter-gatherers* (Doctoral dissertation, University College London).

[CR9] Bowen-Jones E, Brown D, Robinson EJ (2003). Economic commodity or environmental crisis? An interdisciplinary approach to analysing the bushmeat trade in Central and West Africa. Area.

[CR10] Bowles S (2009). Did warfare among ancestral hunter-gatherers affect the evolution of human social behaviors?. Science.

[CR12] Boyette AH, Lew-Levy S, Sarma MS, Valchy M, Gettler LT (2020). Fatherhood, egalitarianism, and child health in two small-scale societies in the Republic of the Congo. American Journal of Human Biology.

[CR11] Boyette AH, Lew-Levy S, Jang H, Kandza V (2022). Social ties in the Congo Basin: Insights into tropical forest adaptation from BaYaka and their neighbours. Philosophical Transactions of the Royal Society B.

[CR13] Bunce, J. A. (2020). Field evidence for two paths to cross-cultural competence: implications for cultural dynamics. *Evolutionary Human Sciences*, *2*. 10.1017/ehs.2020.1 PMC10427313 37588369

[CR14] Bunce JA (2021). Cultural diversity in unequal societies sustained through cross-cultural competence and identity valuation. Humanities and Social Sciences Communications.

[CR15] Bunce JA, McElreath R (2017). Interethnic interaction, strategic bargaining power, and the dynamics of cultural norms. Human Nature.

[CR16] Bunce JA, McElreath R (2018). Sustainability of minority culture when interethnic interaction is profitable. Nature Human Behaviour.

[CR17] Cashdan E (1987). Trade and its origins on the Botletli River, Botswana. Journal of Anthropological Research.

[CR18] Cashdan E (2001). Ethnic diversity and its environmental determinants: Effects of climate, pathogens, and habitat diversity. American Anthropologist.

[CR19] Cashdan E (2001). Ethnocentrism and xenophobia: A cross-cultural study. Current Anthropology.

[CR20] Cochran C, O’Connor C (2019). Inequality and inequity in the emergence of conventions. Politics Philosophy & Economics.

[CR21] Colson E (1979). In good years and in bad: Food strategies of self-reliant societies. Journal of Anthropological Research.

[CR22] Doremus J (2019). Unintended impacts from forest certification: Evidence from indigenous Aka households in Congo. Ecological Economics.

[CR23] Dormitzer PR, Ellison PT, Bode HH (1989). Anomalously low endemic goiter prevalence among Efe pygmies. American Journal of Physical Anthropology.

[CR24] Dounias E, Ichikawa M (2017). Seasonal bushmeat hunger in the Congo Basin. Ecohealth.

[CR25] Duda R, Gallois S, Reyes-Garcia V (2017). Hunting techniques, wildlife offtake and market integration: A perspective from individual variations among the Baka (Cameroon). African Study Monographs.

[CR26] Ensminger J, Knight J (1997). Changing social norms: Common property, bridewealth, and clan exogamy. Current Anthropology.

[CR27] Fa JE, Currie D, Meeuwig J (2003). Bushmeat and food security in the Congo Basin: Linkages between wildlife and people’s future. Environmental Conservation.

[CR28] Fearon JD, Laitin DD (1996). Explaining interethnic cooperation. American Political Science Review.

[CR29] Fry DP, Söderberg P (2014). Myths about hunter-gatherers redux: Nomadic forager war and peace. Journal of Aggression Conflict and Peace Research.

[CR31] Grinker, R. R. (1994). *Houses in the rainforest*. University of California Press.

[CR30] Guernier V, Hochberg ME, Guégan JF (2004). Ecology drives the worldwide distribution of human diseases. PLoS Biology.

[CR32] Handley C, Mathew S (2020). Human large-scale cooperation as a product of competition between cultural groups. Nature Communications.

[CR33] Hewlett BS (1991). Demography and childcare in preindustrial societies. Journal of Anthropological Research.

[CR34] Ichikawa M, Hattori S, Yasuoka H, Reyes-García V, Pyhälä A (2017). Bushmeat crisis, forestry reforms and contemporary hunting among central african forest hunters. Hunter-gatherers in a changing world.

[CR35] Jang H, Boyette AH (2021). Observations of cooperative pond fishing by the Bayaka and Bantu people in the flooded forest of the northern Republic of Congo. African Study Monographs.

[CR36] Joiris DV (2003). The framework of central african hunter-gatherers and neighbouring societies. African Study Monographs Supplementary Issue.

[CR37] Kitanishi K (1995). Seasonal changes in the subsistence activities and food intake of the Aka hunter-gatherers in northeastern Congo. African Study Monographs.

[CR38] Kitanishi K (1998). Food sharing among the Aka hunter-gatherers in northeastern Congo. African Study Monographs Suppl.

[CR39] Kleinschroth F, Laporte N, Laurance WF, Goetz SJ, Ghazoul J (2019). Road expansion and persistence in forests of the Congo Basin. Nature Sustainability.

[CR40] Klieman, K. A. (2003). *“The pygmies were our compass”: Bantu and Batwa in the history of west-central Africa, early times to c. 1900 CE*. Greenwood.

[CR41] Kümpel NF, Rowcliffe JM, Cowlishaw G, Milner-Gulland EJ (2009). Trapper profiles and strategies: Insights into sustainability from hunter behaviour. Animal Conservation.

[CR45] Lew-Levy S, Milks A, Ntamboudila FK, Broesch T, Kline MA (2021). BaYaka adolescent boys nominate accessible adult men as preferred spear hunting models. Current Anthropology.

[CR46] Lew-Levy, S., Milks, A., Kiabiya Ntamboudila, F., Kline, M. A., & Broesch, T. (2022). Costly teaching contributes to the acquisition of spear hunting skill among BaYaka forager adolescents. *Proceedings of the Royal Society B: Biological Sciences*, *289*, 20220164. 10.1098/rspb.2022.0164 PMC9091853 35538787

[CR42] Lewis, J. (2002). *Forest hunter-gatherers and their world: a study of the Mbendjele Yaka pygmies of Congo-Brazzaville and their secular and religious activities and representations* (Doctoral dissertation, University of London).

[CR43] Lewis, J. (2005). Whose forest is it anyway? Mbendjele Yaka pygmies, the Ndoki forest and the wider world. In T. Widlock & W. G. Tadesse (Eds.), *Property and equality, volume 2: Encapsulation, commercialisation, discrimination* (pp. 56–78). Berghahn Books.

[CR44] Lewis J (2008). Ekila: Blood, bodies, and egalitarian societies. Journal of the Royal Anthropological Institute.

[CR47] Lupo, K. D. (2006). In pursuit of the individual: Recent economic opportunities and the persistence of traditional forager-farmer relationships in the southwestern Central African Republic. In B. F. Codding, & K. L. Kramer (Eds.), *Why forage? Hunters and gatherers in the twenty-first century* (pp. 137–166). School for Advanced Research Press.

[CR48] Manson JH, Wrangham RW (1991). Intergroup aggression in chimpanzees and humans [and comments and replies]. Current Anthropology.

[CR49] McElreath R, Boyd R, Richerson P (2003). Shared norms and the evolution of ethnic markers. Current Anthropology.

[CR50] Migliano AB, Page AE, Gómez-Gardeñes J, Salali GD, Viguier S, Dyble M, Vinicius L (2017). Characterization of hunter-gatherer networks and implications for cumulative culture. Nature Human Behaviour.

[CR51] Ministère de l’Economie Forestiere, Republique du Congo (2007). Volume 1: Résumé de la loi 48/83 du 21 avril 1983 définissant les conditions de la conservation et de l’exploitation de la faune sauvage au Congo. Projet WCS Tridom Odzala. Programme Education et Sensibilisation. Available at https://carpe.umd.edu/sites/default/files/publications/4030007_SP_Wildlife_Conservation%26Exploitation_Law_Summary_WCS_2007.pdf.

[CR52] Ministère de l’Economie Forestière et de l’Environnement, Republique du Congo (2010). Le plan d’aménagement de l’unite forestière d’aménagement de Loundoungou Toukoukala. Brazzaville. https://www.openlandcontracts.org

[CR53] Nasi R, Putz FE, Pacheco P, Wunder S, Anta S (2011). Sustainable forest management and carbon in tropical Latin America: The case for REDD+. Forests.

[CR54] Noss AJ (1997). Challenges to nature conservation with community development in central african forests. Oryx.

[CR55] Noss AJ, Hewlett BS (2001). The contexts of female hunting in Central Africa. American Anthropologist.

[CR56] Pisor AC, Gurven M (2016). Risk buffering and resource access shape valuation of out-group strangers. Scientific reports.

[CR57] Pisor AC, Surbeck M (2019). The evolution of intergroup tolerance in nonhuman primates and humans. Evolutionary Anthropology.

[CR58] Pope-Caldwell SM, Lew-Levy S, Maurits L, Boyette AH, Ellis-Davies K, Haun D, Over H, House BR (2022). The social learning and development of intra- and interethnic sharing norms in the Congo Basin: A registered report protocol. PLoS One.

[CR59] Puurtinen, M., & Mappes, T. (2009). Between-group competition and human cooperation. *Proceedings of the Royal Society B: Biological Sciences*, *276*(1655), 355–360. 10.1098/rspb.2008.1060 10.1098/rspb.2008.1060 PMC2581672 18826935

[CR60] Richerson P, Baldini R, Bell AV, Demps K, Frost K, Hillis V, Zefferman M (2016). Cultural group selection plays an essential role in explaining human cooperation: A sketch of the evidence. Behavioral and Brain Sciences.

[CR61] Riddell M (2013). Assessing the impacts of conservation and commercial forestry on livelihoods in northern Republic of Congo. Conservation and Society.

[CR62] Robinson EJ, Barker JL (2017). Intergroup cooperation in humans and other animals. Biology Letters.

[CR63] Rupp, S. (2011). *Forests of belonging: Identities, ethnicities, and stereotypes in the Congo River Basin*. University of Washington Press.

[CR64] Stuart, C., & Stuart, T. (2006). *Field guide to the larger mammals of Africa*. Struik Publishers.

[CR65] Takeuchi, K. (2014). Interethnic relationships between pygmies and farmers. In B. S. Hewlett (Ed.), *Hunter-gatherers of the Congo Basin: Cultures, histories, and biology of African pygmies* (pp. 299–320). Transaction.

[CR66] Terashima, H. (1986). Economic exchange and the symbiotic relationship between the Mbuti (Efe) pygmies and the neighbouring farmers. *Sprache und Geschichte in Afrika*. 7,391–406.

[CR67] Tucker B, Ringen EJ, Tsiazonera, Tombo J, Hajasoa P, Gérard S, Lahiniriko R, Garçon AH (2021). Ethnic markers without ethnic conflict: Why do interdependent Masikoro, Mikea, and Vezo of Madagascar signal their ethnic differences?. Human Nature.

[CR68] Turnbull, C. (1961). *The forest people*. Simon & Schuster.

[CR69] Van Vliet N, Nasi R (2008). Hunting for livelihood in northeast Gabon: Patterns, evolution, and sustainability. Ecology and Society.

[CR70] Wiessner, P. (1982). Risk, reciprocity, and social influences on !Kung San economics. In E. Leacock & R. B. Lee (Eds.), *Politics and history in band societies* (pp. 61–84). Cambridge University Press.

[CR71] Wiessner P (2002). The vines of complexity: Egalitarian structures and the institutionalization of inequality among the Enga. Current Anthropology.

[CR72] Wilkie DS, Carpenter JF (1999). Bushmeat hunting in the Congo Basin: An assessment of impacts and options for mitigation. Biodiversity & Conservation.

[CR73] Wilkie D, Shaw E, Rotberg F, Morelli G, Auzel P (2000). Roads, development, and conservation in the Congo Basin. Conservation Biology.

[CR74] Yasuoka H (2006). The sustainability of duiker (*Cephalophus* spp.) hunting for the Baka hunter-gatherers in southeastern Cameroon. African Study Monographs Supplementary Issue.

[CR75] Yasuoka H (2014). Snare hunting among Baka hunter-gatherers: Implications for sustainable wildlife management. African Study Monographs Supplementary Issue.

[CR76] Zori C, Brant E (2012). Managing the risk of climatic variability in late prehistoric northern Chile. Journal of Anthropological Archaeology.

